# Polydactyly and associated joint dysfunction for left foot: a quintessence case

**DOI:** 10.11604/pamj.2021.39.258.30452

**Published:** 2021-08-20

**Authors:** Tajuddin Burhanuddin Chitapure, Waqar Mohsin Naqvi

**Affiliations:** 1 Mahatma Gandhi Mission School of Physiotherapy, Aurangabad Campus, Mahatma Gandhi Institute of Health Sciences, Navi Mumbai, India

**Keywords:** Polydactyly, bifid finger, ankle and foot, range of motion

## Image in medicine

A 34-year male came to the outpatient department with the complaint of pain in the left leg and foot. The pain was aggravated during walking and standing activities while he felt that ankle and foot joint stiffness was noticeable in the early morning occasionally. On physical examination, there is an extra finger in the left foot know as polydactyly for the left foot which can be a genetic mutation or environmental case with a family history. The range of motion (ROM) for the ankle and foot was full but painful due to tightness present in the left calf muscle. The patient started conventional physiotherapy rehabilitation including ROM exercises, stretching, and strengthening for the calf and intrinsic foot muscles. There was a severe decrease in the pain level from 8 on the Visual Analogue Scale (VAS) to 5 in 8 days of the physiotherapy rehabilitation program.

**Figure 1:**
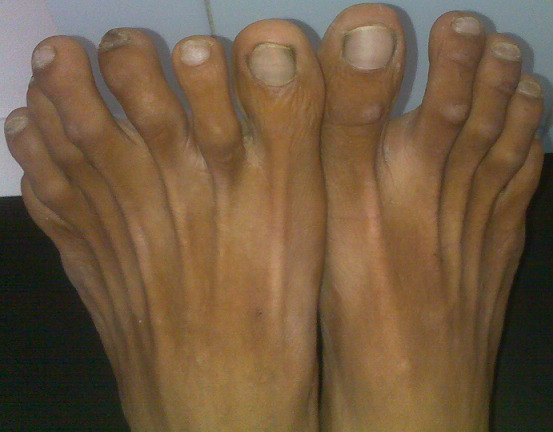
polydactyly of left foot

